# Identification of Starling Circovirus in an Estuarine Mollusc (*Amphibola crenata*) in New Zealand Using Metagenomic Approaches

**DOI:** 10.1128/genomeA.00278-13

**Published:** 2013-05-30

**Authors:** Anisha Dayaram, Sharyn Goldstien, Peyman Zawar-Reza, Christopher Gomez, Jon S. Harding, Arvind Varsani

**Affiliations:** School of Biological Sciences, University of Canterbury, Christchurch, New Zealanda; Department of Geography, University of Canterbury, Christchurch, New Zealandb; The Waterways Centre for Freshwater Management, University of Canterbury, Christchurch, New Zealandc; Natural Hazards Research Centre, University of Canterbury, Christchurch, New Zealandd; Biomolecular Interaction Centre, University of Canterbury, Christchurch, New Zealande; Electron Microscope Unit, Division of Medical Biochemistry, Department of Clinical Laboratory Sciences, University of Cape Town, Observatory, South Africaf

## Abstract

Two complete genomes of starling circovirus (StCV) were recovered from *Amphibola crenata*, an estuarine New Zealand mollusc. This is the first report of StCV outside Europe. The viral genomes were recovered from rolling circle-amplified enriched circular DNA followed by back-to-back primers and specific primer PCR amplification.

## GENOME ANNOUNCEMENT

Circoviruses have circular single-stranded DNA genomes (~1.7 to 2.0 kb) that are encapsidated into icosahedral virons of ~17 to 25 nm in diameter and are known to exhibit strong host specificity, with two species infecting pigs (porcine circovirus 1 [PCV-1] and PCV-2) and the remainder infecting birds (parrots, canaries, ducks, finches, geese, gulls, pigeons, ravens, starling, and swans) (1). Recent isolation of circoviruses and circo-like viruses from dogs (2), fish (3, 4), bat guano (5), and human and primate fecal matter (6) indicates a greater host range than was previously thought. Within New Zealand, three species of circoviruses have been detected: PCV-1, PCV-2 (7, 8), and beak and feather disease virus (BFDV) (9). We report the discovery of two genomes of starling circovirus (StCV), which is the first detection outside Europe.

*Amphibola crenata* (~20) molluscs were collected in 2012 at the Avon-Heathcote Estuary (Christchurch, New Zealand) and were processed as described by Dayaram et al. (10). Viral DNA was enriched by rolling circle amplification (RCA) using the Illustra TempliPhi ampliﬁcation kit (GE Healthcare) as described previously (11–16). The enriched nucleic acid was sequenced at the Beijing Genomics Institute (Hong Kong) using an Illumina HiSeq 2000 (Illumina) platform. The paired-end reads were assembled using ABySS v1.3.5 (17). A preliminary BLASTn (18) analysis of the assembled sequence showed a significant match to an StCV. Further analysis of the contig (2,157 nucleotides [nt]) revealed a complete genome of StCV, sharing 97.5% pairwise identity with the only sequence of starling cirovirus available in GenBank (accession no. DQ172906). To obtain true viral genomes, as the contigs represent a consensus of StCV in the sample, we designed a set of back-to-back primers to recover the StCV genome using PCR. Two sets of primers were designed, one in the capsid protein gene (StCV-CP F, 5′-TTAAGAAGAAGGGGCTGGCTG-3′, and StCV-CP R, 5′-CTTAACAAAATTCATAAGTCTGGCATCA-3′) and the second in the replication-associated protein gene (StCV-Rep F, 5′-GTGAGATCGCGCGAGAGTTC-3′, and StCV-Rep R, 5′-TCATTCCTCTTCCGGCTTTCACAG-3′). The genomes were amplified with the primers using Kapa HiFi HotStart polymerase (Kapa Biosystems), cloned into pJET1.2 (Fermentas) plasmid, and sequenced at Macrogen Inc. (South Korea) by primer walking, and the sequence contigs were assembled using DNAman v7 (Lynnon Biosoft). Both sets of primers recovered full genomes (GenBank accession no. KC846095 and KC846096) that share 99.8% pairwise identity, 99.7% pairwise identity to an assembled contig from an Illumina HiSeq run, and 97.6% pairwise identity to StCV (accession no. DQ172906) from Europe. We did not detect StCV in the tissue of *A. crenata*, indicating it was a “passenger” in the mollusc gut.

The only available sequence of starling circovirus was reported by Johne et al. (19). Limited information is available on the host range and its disease potential in starlings. Starlings were introduced to New Zealand in the mid-1800s and are known to forage in the wrack zone (20) around the estuaries. Given that *A. crenata* molluscs are deposit feeders, grazing on microorganisms and organic detritus on the surface of tidal mudflats (21), they probably also concentrate fecal matter, which might explain the detection of StCV.

### Nucleotide sequence accession numbers.

The two complete genomes of StCV have been deposited at GenBank under the accession no. KC846095 and KC846096.
